# Tai Chi practice and community belonging in active aging: embodied, relational, and place-based embedding in a Beijing community

**DOI:** 10.3389/fpubh.2026.1986641

**Published:** 2026-10-30

**Authors:** Lei Zhang, Xinru Liu, Jinli Jiang, Zeqian Jiang, Fengwei Jia, Guofeng Qu

**Affiliations:** 1 College of P.E. and Sports, Beijing Normal University, Beijing, China; 2 Collaborative Innovation Center of Assessment toward Basic Education Quality at Beijing Normal University, Beijing, China; 3 Beijing Jingshan School, Beijing, China

**Keywords:** active aging, belonging, community participation, internal migration, qualitative case study, Tai Chi

## Abstract

**Background:**

Active aging requires more than preserving physical function; it also depends on meaningful participation, social connection, and valued community roles. Community Tai Chi combines physical activity with opportunities for social participation, yet attendance alone does not establish belonging. This study examined how bodily experience, relationships, and identification with groups and places were connected, and what qualified those connections.

**Methods:**

An interpretive qualitative case study used June 2025 interviews with eight practitioners aged 53–75 years and one community worker in a university-affiliated community in Beijing, China. Grounded theory-informed comparison of edited Chinese texts drew on 95 selected meaning units, 31 subcategories, and eight main categories. Within-participant and cross-participant comparisons retained discrepant accounts.

**Results:**

Participants connected learning and everyday routines with recurring contact, mutual notice, and shared activities. Belonging varied in depth and object: practice acquaintances, team membership, university identification, and residential attachment were distinguishable. New acquaintances did not necessarily imply stronger community cohesion. Only two practitioners (R05 and R08) were identified as internal migrants to Beijing: R05 relocated after retirement for grandchild care, whereas R08 undertook long-term paid domestic care work. Their accounts illustrated opportunities for contact and team belonging, alongside constraints from caregiving and employment. Instruction, volunteer coordination, teaching costs, performance expectations, and institutional permission also qualified participation.

**Conclusion:**

Embodied, relational, and place-based embedding form related interpretive dimensions rather than a demonstrated progression. The contribution is an account of how organized practice offers opportunities for belonging that remain contingent on participants’ circumstances and access to resources. Inclusive provision should consider the fit between practice arrangements and everyday responsibilities, distinguish the objects of belonging, and evaluate organizational changes separately from health outcomes. Shared skill as a portable social credential remains a provisional explanation of initial contact, not evidence of durable migrant inclusion.

## 1 Introduction

Active aging is shifting the work of public health beyond disease treatment toward the maintenance of functional ability, meaningful participation, and dignified everyday life. The World Health Organization’s active aging framework places health, participation, and security within a shared policy horizon, while the healthy aging agenda emphasizes the interaction between intrinsic capacity and supportive environments rather than the absence of disease alone (1, 2). This shift gives ordinary community activities public health significance: the relevant question is not whether they sit inside or outside a clinical system, but whether they offer sustained opportunities for movement, relationships, and valued social roles. With global population aging accelerating, community-based preventive practices have become increasingly important (3). Participation in community activity groups has been associated with physical activity, perceived health, and well-being, and community physical activity may be linked to subjective well-being through social capital and intergenerational support (4, 5). These associations leave an important question for qualitative inquiry: how do participants connect an activity with relationships and belonging, and where do those connections remain limited?

This question is particularly relevant when retirement, family care, or employment changes the organization of daily life. In China, internal migration can also require people to establish local contacts while negotiating household registration, work, caregiving, and institutional access (6–8). National studies suggest that community health education is associated with self-rated health and social integration among older migrants, while formal coverage and accessible participation routes remain incomplete (8, 9). Recent research similarly shows that later-life resocialization is shaped jointly by family support, policy satisfaction, community development, and personal resources (10). However, having a migration history, moving in later life, and moving between activity sites describe different circumstances. A study of a community group must establish which of these circumstances its participants actually report before making claims about migrant inclusion.

Tai Chi offers a culturally specific setting in which to examine the connections between community practice and belonging. It is simultaneously a traditional martial art, a mind–body exercise, and a public cultural practice, with limited equipment requirements and high visibility in parks, campuses, and residential neighborhoods (11). Community programs, qualitative studies, reviews, and cross-sectional research have linked Tai Chi participation with balance, lower-extremity function, sleep, cognition, psychosocial well-being, and perceived health (12–17). Research on related traditional exercises also evaluates balance and cognition among community-dwelling older adults (18). Much of this literature focuses on health outcomes or adherence. Less is known about how the practical work of learning and sustaining community Tai Chi is connected with different forms of belonging.

This individual-outcome bias is not unique to Tai Chi research. Recent active and healthy aging scholarship has conceptualized integrated arts engagement as preventive infrastructure, arguing that cultural participation can support function, meaning, and social connection when it is accessible, continuous, and connected to local partnerships (19). Likewise, conceptual accounts of cultural participation as preventive infrastructure invite attention to accessible provision without demonstrating the effectiveness of every activity (20). We therefore examine participants’ descriptions of contact, mutual notice, and identification with particular groups or places, rather than using attendance as a proxy for integration.

Research on sport and later-life migrant integration has begun to address this problem directly. Sun and Wang developed a grounded theory model from 34 aging migrants, linking social inclusiveness, intergenerational relationships, social networks, social memory, psychological integration, and identity recognition, while acknowledging that sport is not a universal solution (21). The present study examines a community Tai Chi team and people connected to its activities. Only R05 and R08 were identified as internal migrants to Beijing, with family caregiving after retirement and long-term paid domestic care work providing distinct migration contexts. These accounts inform exploratory comparison within the community case. Research on group exercise also identifies instruction, social support, and program organization as relevant to participation (22–24). The setting and organization of practice therefore merit examination alongside the characteristics of Tai Chi itself.

We use embedding as an interpretive term for how practice is situated in everyday routines, relationships, and settings. Embodied embedding concerns learning, perceived capability, and the organization of daily activity. Relational embedding concerns recurring contact, mutual assistance, and membership of the practice group. Place-based embedding concerns attachment to settings. In examining the latter, we distinguish residential community attachment from identification with a university and from belonging to a team that may practice in several places. Weak-tie theory helps interpret recurring acquaintances (25), while place-attachment scholarship provides a basis for considering person–place bonds involving individual and collective meanings, affective, cognitive, and behavioral processes, and both social and material environments (26, 27). Research on later-life roles further directs attention to participants’ different expectations of involvement (28). These concepts inform interpretation rather than define measured outcomes or predetermined stages.

Against this background, we asked how practitioners describe entering and sustaining community Tai Chi, how bodily practice is connected with relationships and different objects of belonging, and which organizational conditions qualify those connections. The aim of this study is to develop an evidence-based account of this community case and identify implications for inclusive participation in active aging. Migration accounts contribute exploratory comparisons within this aim; the study does not estimate migrant integration or a Tai Chi effect.

## 2 Materials and methods

### 2.1 Study design and methodological positioning

We conducted an interpretive qualitative case study of one community Tai Chi team, including its connections with other practice sites and community administration. The case-study design defines the setting and scope of the inquiry. Grounded theory-informed procedures were used to compare and organize an already assembled interview corpus: open coding identified meanings in selected passages, axial comparison related categories to conditions and activities, and integrative coding developed an explanatory account. This use of procedural coding and constant comparison draws on Strauss and Corbin, with attention to co-constructed meaning and researcher position informed by Lou et al. (29) and Strauss and Corbin (30). It is a retrospective analysis rather than a full grounded theory design involving concurrent theoretical sampling.

### 2.2 Case setting

The team was based in a university-affiliated residential community in Beijing. Activities included routine practice, specialist teaching, preparation for public events, and contact with a district association and community administration. Interviewees described seasonal morning schedules, with some evening or weekend participation. R05 mainly practiced at another site and joined selected team events. Routine practice and invited specialist lessons involved different arrangements: R02 valued optional paid instruction, whereas R04 raised concerns about fees and continuity. The team leader described welcoming employees, retirees, relatives, care workers, and students. Access to university facilities nevertheless depended on institutional permission. The case, therefore, encompasses different degrees and forms of involvement, rather than a standardized program with a uniform attendance or fee policy.

### 2.3 Participants and sampling

Sampling was purposive and drew on practitioners with continuing experience of community Tai Chi who were willing to discuss it, together with a community worker. The nine interviews represented variation in age, gender, occupational background, practice experience, and organizational role (Table 1). The first author’s access began when a senior colleague who taught the community team invited him to supplement its competition squad for a Haidian District community Tai Chi competition. Through shared training and competition, he met the team leader. Interview recruitment subsequently began with the leader: some participants were contacted or recommended by her, and others were approached by the first author during routine practice. These routes favored established, cooperative participants and may have underrepresented less connected or dissatisfied members. A comprehensive record of the numbers approached and declining interviews was not available in the retained study materials; a recruitment response rate cannot be reported.

**Table 1:** Participant characteristics, migration context, and involvement in the focal team (*N* = 9).

ID	Gender	Age (years)	Occupational background	Migration context	Tai Chi experience	Role or involvement
R01	Woman	62	Retired university administrator	Not classified as a migrant	About 10 years, intermittent; regular focal practice in past year	Team leader; association organizer
R02	Woman	57	Early-childhood education/management	Not classified as a migrant	About 7 months; joined October 2024	Beginner; writing and publicity
R03	Woman	70	Retired technical-standards worker	Not classified as a migrant	Intermittent prior learning; about 2 months in focal team	Self-directed learner
R04	Man	75	Retired university logistics manager	Not classified as a migrant	About 1 year; joined March 2024	Early member
R05	Man	62	Retired school teacher	Internal migrant from Hunan; relocated after retirement for grandchild care	About 6 years; occasional focal-team events, usual practice elsewhere	Family-care responsibilities; cross-site participation
R06	Man	70	Retired university technical manager	Not classified as a migrant	About 1 year; joined June 2024	Peer-recruited; expertise advocate
R07	Woman	58	Long-term family caregiver	Not classified as a migrant	About 3 years; practiced with current leader since 2024	Core member; participation across social settings
R08	Woman	53	Domestic care worker; 16 years in Beijing	Internal migrant; long-term paid domestic care work in Beijing	About 11 years total; focal-team events since 2024	Core member; publicity and organizing
R09	Woman	30	Community worker	Not classified as a migrant	No Tai Chi practice; governance connection	Governance perspective

Ages, occupational backgrounds, and approximate total practice durations follow the participant information in this table. Migration context concerns the classification used in this study: only R05 and R08 are identified as internal migrants to Beijing; R02 and the other participants are not classified as migrants. This classification does not establish a complete lifetime residential history for every participant. R05 moved from Hunan after retirement to help care for a grandchild while his adult child worked in Beijing; R08 undertook long-term paid domestic care work in the community. Their interviews do not provide equally detailed relocation chronologies. Total Tai Chi experience and involvement in the focal team are reported separately; in particular, R08’s approximately 11 years of total experience differs from her involvement in focal-team activities since 2024. Roles describe participation, not measured inclusion. Participant codes replace names.

The eight practitioners were aged 53–75 years; the community worker was 30. Only R05 and R08 were identified as internal migrants to Beijing. R02 is not classified as a migrant and is not included among the migration cases. R05 was a retired teacher from Hunan who moved to Beijing after retirement in 2023 to help care for a young grandchild while his adult child worked there. His age at relocation was approximately 60, inferred from his birth year. R08 was a 53-year-old domestic care worker who reported 16 years in Beijing and long-term paid care work in the community. Her interview did not establish an exact relocation date or directly elicit her age at relocation, and her move is not characterized as later-life migration. These family-care and employment contexts are distinguished from movement between Tai Chi practice sites. The two accounts support exploratory comparison, not a uniform or representative migration pathway.

Eligibility was based on direct experience of team activities or community governance, willingness to be recorded, and consent to anonymized quotation. The sample permits focused comparison of an organizer, practitioners with different involvement and responsibilities, and a governance perspective. Its adequacy is considered in relation to this bounded aim, following information-power reasoning (31). It includes no direct accounts from leavers, people declining to join, or rejected applicants. The coding record describes analysis of R01–R06, R08, and R09 followed by a check against R07. Repetition in that check was not a prospective recruitment stopping rule or evidence of saturation of migrant experiences.

### 2.4 Data collection

The first author conducted the interviews, drawing on prior shared training and competition with team members. The community’s proximity to the university campus facilitated access and the arrangement of interviews. Semi-structured interviews took place between June 11 and 26, 2025, in quiet community or campus settings. Topics included entry into Tai Chi practice, perceived bodily changes, learning, relationships, organization, public activities, and resources; mobility was discussed where relevant. Appendix A summarizes the questions recorded in the interviews and is a retrospective topic guide. Recording and initial transcription used Feishu’s recording and transcription functions. The second and third authors reviewed and processed the resulting Chinese text, removing colloquial or repetitive elements to prepare it for coding. The study records report interviews of approximately 23–82 min and a corpus of approximately 52,000 Chinese characters. The analyzed texts retain questions and responses but are edited transcripts rather than unmodified verbatim transcriptions.

The first author’s participation in practice, competition preparation, and public activities informed his understanding of the setting and movement terminology. This experience supplied context rather than a separately coded observation dataset. Interview accounts are the empirical source for reported events, online communication, and organizational arrangements. Observation and messaging-group material are not used as independent longitudinal evidence.

### 2.5 Data analysis

Comparison proceeded within participants’ accounts and across participants, using source locations, labels, category descriptions, and analytic memos. Axial comparison related entry circumstances, learning, and organization to reported relationships and identifications, while examining constraints and discrepant accounts, while examining constraints and discrepant accounts. The integrative account organized these comparisons through embodied, relational, and place-based embedding. The coding record documents these terms in the final synthesis but does not establish a dated sequence for their first introduction. We therefore present them as an interpretation informed by empirical comparison and sensitizing literature, rather than claiming wholly inductive emergence. Table 2 summarizes the categories; Appendix B provides the subcategories and worked examples linking evidence, initial labels, and revised interpretations.

**Table 2:** Coding categories and their role in the interpretation.

Main category	Subcategories summarized	Interpretive use	Selected units
A Entry circumstances and activity fit	Health risk/recovery; retirement and caregiving transitions; activity fit and aesthetic choice	Entry conditions	12
B Reported embodied experience	Perceived bodily change; attention and emotion; routine and competence; self-presentation	Embodied interpretation	8
C Skill learning and instruction	Beginner difficulty; demonstration/correction; video reflection; event feedback	Conversion condition	9
D Organization and expectations	Volunteer governance; regular rhythm; mutual-help norms; learning–event cycle	Organizational or learning condition	12
E Social ties and participation across settings	Practice acquaintances; contact across sites; family and work-network diffusion	Relational interpretation	14
F Resources and institutional coordination	University expertise; association networks; community/subdistrict projects; coordination	Institutional interface	11
G Cultural expression and collective identification	Cultural interpretations; aesthetics; visibility; pride and branding plans	Place-related interpretation	11
H Constraints on participation and continuity	Space/season/noise; age/gender/time; injury; resource/leader dependence; scale	Boundary conditions	18

Each selected unit has one primary category. Counts concern the retained coding record and are not exhaustive counts of relevant passages or participants endorsing a finding. Additional contextual checks are documented in Appendix C without changing this 95-unit total.

Reanalysis returned to all nine full texts to check whether the selected summaries preserved timing, speaker, and context. Comparisons corrected R05’s learning-before-relocation chronology, distinguished R08’s team belonging from limited contact at another site, and retained R07’s acquaintance alongside her rejection of a cohesion claim. Reports of another person’s injury, community branding, and possible growth were separated from firsthand experience. These decisions are documented in Appendices B, C. Unit counts describe the retained record and were not used to rank mechanisms or demonstrate saturation of migrant experiences.

Coding and comparison used Chinese texts. Quotations were selected to make both the interpretation and its limits visible, with English renderings compared with the edited Chinese text and surrounding passage using DeepL and WPS translation tools. Compressed coding summaries are presented as paraphrases. For example, 拳友 is rendered as “practice friends,” while 找到了一个组织的感觉 is rendered as “a feeling of having found a group to belong to.” Neither expression alone establishes residential integration. Appendix C presents selected English renderings and the interpretive decisions they support. This text-based process is not reported as independent bilingual-expert validation, formal back-translation, or a complete audio audit.

### 2.6 Reflexivity and rigor

The first author’s shared training and competition with participants helped elicit technical and organizational detail but could encourage deference, socially desirable responses, or an emphasis on successful participation. Leader-mediated referrals could reinforce that tendency. Some interview questions invited agreement about harmony, inclusion, or the value of teaching, and the learning–practice–competition–performance cycle was partly formulated within interviewer–participant exchanges. During reanalysis, interviewer formulations were distinguished from participants’ accounts, quotations were read with surrounding qualifications, and favorable descriptions were compared with disagreement, selective attendance, evaluation pressure, and R07’s rejection of a community-cohesion claim. The first and second authors’ involvement in analysis and the second and third authors’ transcript review are reported as collaborative tasks; they do not establish independent coding or an inter-coder agreement test. No formal member checking was reported. These checks informed the explanation but could not eliminate the influence of prior relationships or recruitment. Reporting was guided by COREQ and attention to credibility, traceability, and transferability (32). Procedural grounded theory reporting in established Chinese education studies was also consulted as a technical reference for category formation, core categories, and feedback cycles (33, 34).

Generative artificial intelligence was used during manuscript preparation for structural reorganization, language editing, and Chinese–English translation. The analysis was grounded in the original interviews, coding workbook, and analytic memos supplied and reviewed by the authors. AI did not replace informed consent, data collection, or author responsibility for validating codes, quotations, references, interpretations, and conclusions.

### 2.7 Ethical considerations

The study was approved by the Institutional Review Board of the College of Physical Education and Sports, Beijing Normal University (approval number BNUTY2025-060601; June 6, 2025), and was conducted in accordance with the Declaration of Helsinki and applicable institutional requirements.

Participants were informed about the study purpose, recording, quotation, and intended use of the interviews and provided written informed consent. Codes R01–R09 are used throughout. Table 1 retains ages and broad occupational backgrounds needed to assess the population and practice histories. Names, exact employers, unnecessary site identifiers, and third-party identifiers are omitted. Combinations of contextual details can nevertheless identify people in a small community, so sensitive health histories are summarized only as needed for interpretation. Full interview transcripts and recordings are not publicly available; any additional access to redacted excerpts is subject to consent, ethical requirements, and assessment of re-identification risk, as specified in the Data availability statement.

## 3 Results

### 3.1 Overview of the interpretive framework

The eight categories summarized in Table 2 connect entry circumstances, bodily experience, learning, organization, relationships, resources, cultural expression, and constraints. Counts describe the distribution of the 95 selected units in the coding record, rather than the prevalence or importance of a finding. Appendix B lists all 31 subcategories, and Appendix C presents selected English renderings and the interpretive decisions they support.

The framework distinguishes related dimensions of embedding rather than ordered stages (Figure 1). Learning, perceived capability, and routine describe embodied experience; recurring ties and team membership describe relational involvement; and place-related accounts require attention to their specific referent. Table 3 compares all nine interviews, including relocated members and other practitioners. Bodily engagement, new acquaintances, and team pride could occur without an expressed increase in residential attachment. The comparison, therefore, supports a conditional account of belonging: organizational arrangements make contact possible, but the depth and object of belonging depend on how people can and wish to participate. Missing or qualified accounts are not treated as proof that an outcome was absent.

**Figure 1:**
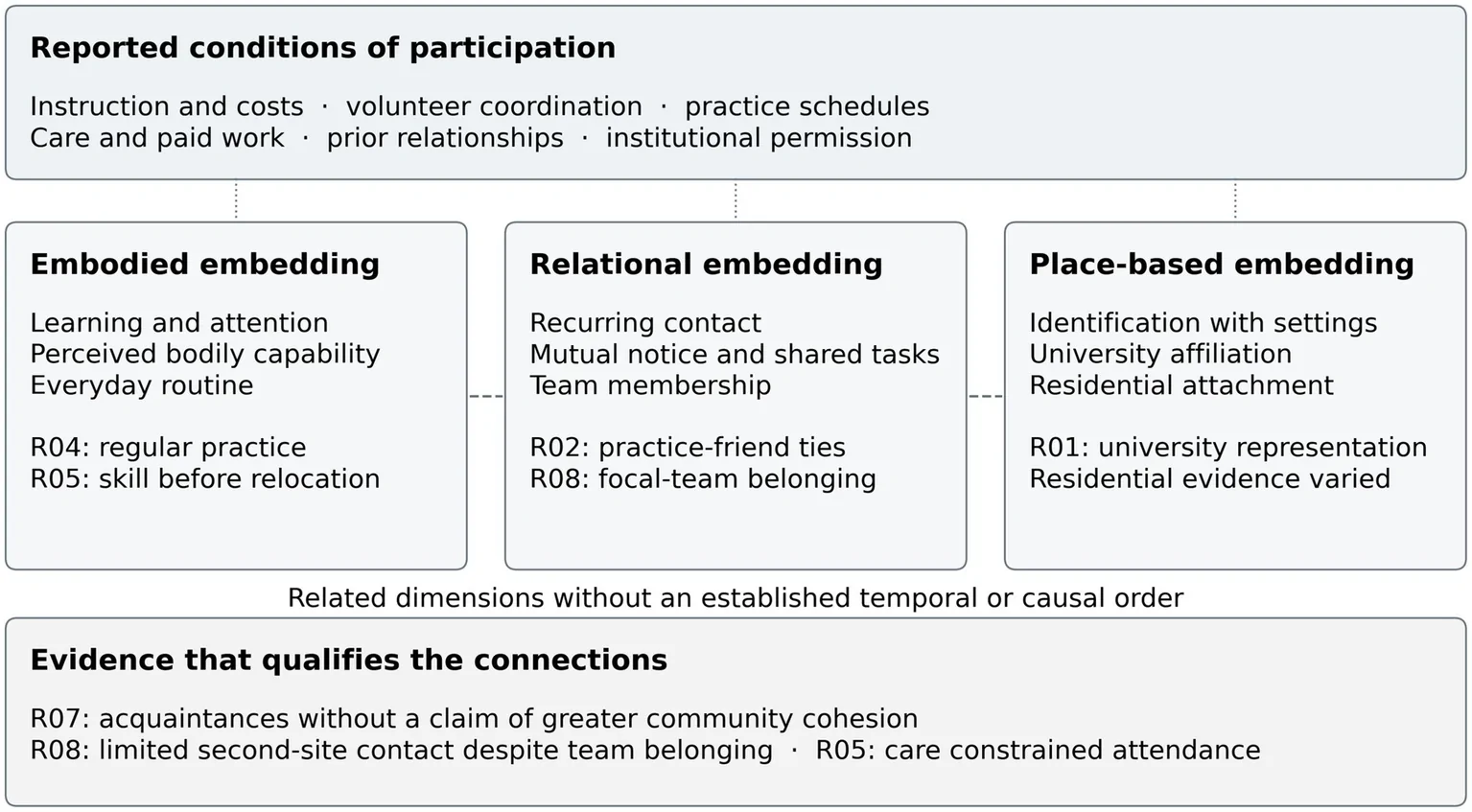
Related embedding dimensions and the conditions of belonging in community Tai Chi. The panels summarize bodily experience, relationships, and identification with settings; they are not temporal stages. Participant examples identify both supportive and incomplete connections. Dashed links indicate interpretive connections without a required order or established causal direction. Team membership, university identification, and residential attachment remain distinct. Conditions and constraints were reported in interviews; the figure does not depict measured health outcomes, verified feedback, or a general migrant-inclusion effect.

**Table 3:** Evidence for embedding dimensions and incomplete connections across participants.

ID	Embodied experience and involvement	Relationships and object of belonging	Conditions and incomplete connections
R01	Learning connected with teaching and voluntary organization	Team pride; representing the university	Institutional identification and organizing experience preceded current team; selective value expectations
R02	Instruction, routine, intensive event preparation	Practice friends, mutual notice, university pride	Not classified as a migrant; beginner learning and practice ties; anxiety, teaching costs, and criticism of provision
R03	Solitary learning followed by about 2 months with the team	New acquaintances and appreciation of broader community activities	Values solitude; community appreciation not attributable solely to Tai Chi
R04	Health-related search for regular practice; stable routine	Existing colleague recruited; team participation and public events	Declined difficult new routine; no demonstrated change in residential identity
R05	Learning in 2019 preceded the move in 2023; mainly another site	Park-group entry; occasional representation of the university team	Relocation from Hunan for grandchild care; family affiliation and event contacts; care schedule limited focal-team attendance
R06	Existing colleagues repeatedly encouraged entry; valued teaching	Public team recognition but few reported new friendships	Prior university ties; technical disputes and fear of evaluation
R07	Preferred concentration alone, persistence with others	New acquaintances and some trusted exchanges	Explicitly rejected a direct increase in community cohesion
R08	Longer total experience; work change enabled current involvement and self-reported relaxation	Focal-team belonging; care-worker ties; brief contact at another site	Internal migrant undertaking paid domestic care work; prior work networks and work schedule; relocation age not directly elicited
R09	Not a participant in practice	Reported program support and planned community branding	Governance perspective and aspirations do not measure residents’ belonging

Entries summarize participant accounts, not ratings of achieved integration. Only R05 and R08 are identified as internal migrants in this study; R02 is not classified as a migrant. R05’s relocation for grandchild care and R08’s long-term paid domestic care work are distinct contexts, and their relocation chronologies were not elicited in comparable detail. Unreported links are not coded as absent outcomes.

### 3.2 Entering practice through changing needs and opportunities

Health concerns, life transitions, and perceived suitability provided different reasons for beginning Tai Chi. R02 sought an activity compatible with ongoing discomfort, whereas R04 searched for regular group practice after a serious health episode. R06 combined a wish to remain active with repeated encouragement from a former colleague. Health was therefore part of the context of entry, alongside existing relationships and the availability of an acceptable activity.

Retirement and care responsibilities changed the time available for practice. R03 sought movement and social contact after retirement. R05 began systematic Tai Chi in Hunan in 2019, when his spouse’s move to help care for a grandchild made partner dancing less convenient. He moved to Beijing after retiring in 2023 to help care for a young grandchild while his adult child worked there; prior skill subsequently offered a way to approach other practice groups. R08’s situation involved long-term paid domestic care work in Beijing. She described gaining personal time when she changed from live-in infant care to daytime care work: “Now I can think about myself a little more.” Her account concerns a change within continuing employment, rather than retirement. Family caregiving and paid care work therefore shaped participation in different ways.

Participants also compared Tai Chi with other activities. R07 preferred movement without running or jumping, and R03 valued being able to practice “with the group or alone.” Yet perceived suitability did not remove the work of learning complex sequences or fitting practice around care and employment. The same activity could be attractive while remaining difficult to attend regularly.

### 3.3 Embodied embedding in learning and everyday routines

#### 3.3.1 Learning difficult movements

Learning required practitioners to coordinate movement, remember sequences, and judge their own performance. R06 initially doubted that a group of older practitioners was doing more than “going through the motions.” He struggled because “when my hands caught up, my feet did not; when my feet caught up, my hands did not.” Demonstration and detailed explanation changed his appraisal of the activity. R02 likewise valued specialist instruction because, as a beginner, she could learn without first unlearning a familiar routine. In these accounts, instruction made participation more understandable and worth sustaining.

Participants used several ways of learning. R03 combined explanations, replayed demonstrations, and individual experimentation. R08 recalled a disagreement about a fan movement that was clarified after a specialist demonstration. Teaching could therefore provide a shared reference for discussing technique, although R06 also described disagreements among members. Access involved practical choices: R02 regarded optional lessons as worth paying for, while R04 questioned fees and the continuity of paid teaching.

#### 3.3.2 Perceived bodily and emotional changes

Participants evaluated practice through everyday comparisons. R02 perceived better coordination and concentration; R05 compared his current ability to stand on one leg with his earlier performance; R08 felt that her body had “opened and stretched” and that she slept more soundly; and R04 described more regular eating, sleep, and daily routines. These were perceived changes recalled in interviews. Some participants also practiced other traditional exercises, and no standardized health assessments were available to attribute change to Tai Chi.

Attention to movement was also connected with emotional experience. R02 described a more balanced state of mind, R05 said he could settle down, and R08 recalled becoming absorbed during solo practice. R07 distinguished the concentration possible when practicing alone from the persistence she associated with practicing alongside others. Her account suggests that solitary and collective practice met different needs, rather than representing successive levels of engagement.

#### 3.3.3 Routine and self-presentation

Practice provided a recurring structure in some participants’ lives. R04 treated it “like going to work” and notified the leader when absent. R05 described early rising and an established practice habit that preceded his move. Public events offered additional learning goals: R02 undertook intensive rehearsal for a demonstration, although the commitment also caused anxiety. R07 valued awards and hoped to pursue an instructor qualification, whereas R03 and R06 did not seek such roles. Competence and recognition had different meanings for different members.

Clothing, music, movement, and images were part of how participants presented themselves. R01 described the uniformly dressed team as “a moving landscape.” R05 found it easier to enter the movement state when dressed in Tai Chi clothing and noted that synchronized fan sounds and formations drew passers-by. R06, by contrast, avoided conspicuous dress because he feared a mismatch between his appearance and skill. Public visibility could be enjoyable while also creating pressure to appear competent.

### 3.4 Relational embedding through recurring contact

#### 3.4.1 Practice schedules and volunteer coordination

R02 connected persistence with a regular group: “If it were just me, I would never keep it up.” She also described peers noticing absence and asking after one another. Attendance remained flexible: R03 emphasized being free to stay home when tired or occupied, and R05 and R08 fitted practice around care or work. Recognizable routines provided occasions for contact without implying identical attendance patterns.

Continuity involved organizational work. R01 prepared teaching, coordinated events, contacted specialists, applied for projects, and organized publicity. R04 valued this commitment. R02 also described another member leading practice when R01 was absent and others circulating notices. The accounts show concentrated volunteer responsibility with other members providing cover when needed, rather than complete dependence on one person.

#### 3.4.2 Practice acquaintances and mutual notice

Regular contact did not necessarily become close friendship. R02 explained, “I would not call them friends; I would call them practice friends.” Although conversation centered on practice, members noticed illness, medical appointments, and unusual absences. R03 valued solitude but also welcomed opportunities to speak with others after retirement. Such ties offered companionship and recognition without requiring intimate disclosure.

R07 described meeting people she had not known before and occasionally discussing worries with trusted peers. Yet, when asked whether Tai Chi improved community cohesion, she replied, “No; I just come to practice.” R06 also reported few new friendships despite participating in public events. These accounts qualify the connection between repeated interaction and wider belonging: acquaintance and selective support could coexist with little perceived change in community cohesion.

#### 3.4.3 Contact across practice sites

R05, a retired teacher from Hunan, connected prior Tai Chi skill with entry into practice groups after moving to Beijing for grandchild care. Learning in 2019 preceded his own relocation in 2023. Describing an encounter with a park group, he recalled, “I asked, ‘May I join you?’ They said yes,” and explained, “I feel that knowing a little Tai Chi helps me find friends quickly.” This account supports his perception that a shared activity helped initiate contact; it does not establish that skill was tested or required for admission. His usual practice was at another site, where an earlier schedule fitted family tasks, and he joined selected focal-team events through competition contacts and a family connection to the university. R02’s account contributes evidence about beginner learning and practice acquaintances, rather than a migration narrative.

R08, an internal migrant undertaking long-term paid domestic care work in the community, described belonging in the focal team: “It feels like I have found a group to belong to.” She also reported informal practice with care-worker peers and explained, “At least now I have a little free time of my own in the mornings and evenings, so I practice more.” These accounts indicate how practice could offer contact and valued personal time within continuing employment. Her contact at a second site near work was more limited: “I have not really joined their group; I just watch and copy some movements.” She explained that work took priority. Her account distinguishes focal-team belonging, contact at another site, and participation through established work ties; it does not establish unrestricted or durable integration into the wider residential community.

Across these accounts, new contacts and existing relationships both mattered (Table 3). R03 met practice acquaintances while continuing to value solitude; R06 entered through a former colleague and reported few new friends. R05’s prior skill and family affiliation supported opportunities that his care schedule limited. R08 combined focal-team belonging with work-constrained contact elsewhere. These differences led us to treat contact, membership, and wider community attachment as separable experiences.

### 3.5 Team pride and identification with different places

Public events made affiliation visible, but the object of identification varied. R01 described preparing to represent the university and taking pride in collective achievement. R02 associated a routine with its university origin, and R06 recalled compliments directed at the university team. Several participants already had university connections, so these accounts may express or reinforce an established affiliation. They provide clearer evidence of team pride and university identification than of newly developed attachment to the residential community.

Practice experiences also circulated beyond the immediate group. R05 shared short videos with contacts elsewhere, R02 wrote reflections, and R08 sent videos to her children. These were ways of presenting an active life and maintaining conversation. The interviews provide no corresponding account of measured recruitment or changes in residential belonging attributable to online circulation.

R09 described plans to develop Tai Chi into a recognizable community program and seek wider recognition. This was a governance aspiration. R01 reported project support and considered team quality relevant to further applications, while R09 discussed assessment criteria. Their accounts suggest a perceived connection between recognition and resources. Members nevertheless appraised community provision differently: R03 valued its broader range of activities, whereas R02 questioned how administrative support translated into personal benefit. Team identification could coexist with criticism of community provision.

### 3.6 Instruction and organizational resources

Specialist instruction was valued both for learning and for reducing uncertainty. R06 associated correction with subsequent relief from knee discomfort, and R07 sought guidance partly because she feared injury. These accounts explain why instruction mattered to participants; they do not establish a clinical preventive effect. The contrasting views of R02 and R04 about paid lessons also show that the value of instruction and its affordability or acceptability were separate questions.

Learning, routine practice, competitions, demonstrations, and communication were linked in several narratives. R02’s event deadline prompted rehearsal, R04 described invitations to perform, and R01 discussed specialist sessions and project applications. The formulation of a recurring activity cycle was partly developed through interviewer prompting and is treated as an analytic summary. Event preparation offered opportunities for involvement while also generating pressure and selective refusal. The interviews did not establish a feedback effect from awards or visibility to subsequent recruitment and funding.

Reported support came from university staff and students, association contacts, and community administration. It included teaching, publicity, clothing, transport, and project funds. R04 and R09 emphasized that particular facilities or resources still depended on permission and coordination. The accounts make institutional arrangements part of the explanation of participation, even where expertise and facilities were nearby.

### 3.7 Constraints and discrepant accounts

#### 3.7.1 Shared space and institutional access

R09 described limits on the community’s authority over university-owned facilities: “We can only apply.” The community worker also reported that residents without a university employment connection could encounter difficulties entering campus. R04 considered some nearby rooms underused but difficult to obtain. These accounts distinguish physical proximity from permission to use resources, while leaving participants’ individual access rights unverified.

Practice space was shared with dance, calligraphy, and other activities and was close enough to homes to generate noise complaints. Participants also described winter conditions, rain, snow, and summer heat limiting outdoor practice. Availability, therefore, depended on ownership, schedules, competing uses, and neighborhood relations as well as the existence of an open space.

#### 3.7.2 Time and participation demands

Care and work limited when some practitioners could attend. R05 preferred an earlier practice group because the focal team’s schedule conflicted with shopping and cooking. R08 used evenings, weekends, or spare time around employment. R06 initially resisted joining partly because of his impression that the group consisted mainly of older women. These are accounts of perceived barriers among people who eventually participated; people unable or unwilling to join were not interviewed.

#### 3.7.3 Safety concerns and informal boundaries

Safety narratives differed in evidentiary status. R06 recalled his own knee discomfort and later improvement following instruction; R07 described fear of injury without reporting that she had sustained it. R08 recounted a care recipient’s knee problem and his attribution of it to intensive practice. That second-hand explanation was not clinically assessed. R04 provided a more direct account of limiting demands: “They started learning another new routine, but I did not join in. I could not manage more than 50 new movements.” Selective refusal was compatible with continued membership.

Expectations about skill and contribution could also complicate inclusion. R06 feared public evaluation, and R02 expressed aesthetic and cultural distinctions between Tai Chi and other activities. These are participants’ judgments, not an objective hierarchy of activities or people. R01 welcomed different occupational groups while preferring members who shared the team’s values and contributed willingly. This suggests a possible informal boundary, although no rejected applicant was interviewed. R09’s comparison of managing 20 and 200 people was similarly a warning about possible future demands, not evidence of an observed expansion or organizational breakdown.

## 4 Discussion

### 4.1 Principal findings

This case connects bodily learning and routine with opportunities for recurring contact, while showing that participation had different meanings for different practitioners. Its main contribution is an account of conditional connections across embodied, relational, and place-based experience. Forming practice acquaintances, belonging to a team, expressing university affiliation, and feeling attached to a residential community were distinguishable. R02’s practice-friend ties, R07’s new acquaintances without an endorsement of greater cohesion, and R08’s contrasting involvement across sites make those distinctions empirically consequential. They identify where an account of progression from practice to community belonging would exceed the evidence. Figure 1 and Table 3 bring these connections and limits together.

### 4.2 Embodied practice within organized embodied activity

Research on balance, lower-extremity function, sleep, cognition, and psychosocial well-being provides a plausible context for participants’ bodily narratives (12–18), but self-reported change in this study cannot establish clinical efficacy. The present interviews add detail about how such experience became meaningful in everyday practice: instruction provided a reference for judging movement, repeated attendance structured time, and events made competence visible. These features help explain why an exercise activity could also become a valued routine and a setting for interaction. At the same time, solitary practice remained meaningful, and specialist teaching, public performance, and leadership were not equally desired by all participants.

### 4.3 Shared skill and opportunities for initial contact

The recurring acquaintances described by R02 and R03 are consistent with the limited but meaningful support associated with weak ties (25). Other contacts involved extended family members and people connected through work or the university. R05 and R08 additionally described approaching practitioners at unfamiliar sites, although the depth and continuity of those encounters differed. This distinction matters for the proposed portable social credential: the accounts support a question about how shared skill enables initial contact, not a conclusion that it produces lasting inclusion.

We use portable social credentials as a provisional metaphor for the situational recognition of shared practical competence that may help initiate interaction with unfamiliar practitioners. In relation to Bourdieu’s distinction between embodied cultural capital and resources available through social relationships, the proposal concerns a possible use of acquired competence at the point of contact, rather than a new form of capital (35). It also differs from social identity, which concerns self-definition through group membership (36), and from participation, which describes involvement in activities at different levels of interaction and contribution (19). R05 associated knowing Tai Chi with approaching a group, but his account does not show that skill was assessed or required for admission. A welcoming group, prior contacts, or general sociability may explain access. R08’s limited second-site involvement further shows why initial recognition and sustained membership require separate evidence. Future comparison should examine whether competence changes how unfamiliar newcomers are received; equally accessible entry for novices would weaken a credential-specific explanation.

### 4.4 Institutional affiliation and place attachment

Place attachment is not a static feeling toward physical space. It develops through individual and collective experience, affective, cognitive, and behavioral processes, and interactions with social and material environments (26, 27). This distinction changes the interpretation of public representation in the present case. Representing a university could express existing affiliation, while belonging to a team could remain meaningful across different practice sites. Neither necessarily indicates a stronger bond with the residential community. R03’s appreciation of wider community activities, R02’s criticism of provision, and R07’s reservation about cohesion further demonstrate variation in what the locality meant to participants.

The governance accounts also show why opportunities cannot be inferred from nearby resources alone. Institutional permission and coordination affected access to facilities and teaching, including within a setting with strong university connections. This finding complements sport and migration research on the social conditions of inclusion (21). It does not show that participation overrides institutional status: opportunities for friendly contact and restrictions on access can coexist. R09’s proposed community brand is consequently interpreted as an intended organizational outcome, rather than evidence of an achieved change in residents’ belonging.

### 4.5 Alternative explanations and transferability

Tai Chi contributes recognizable movement forms, opportunities for technical learning, bodily comparison, and cultural expression to this case. Recurring meetings, committed organizers, shared tasks, and public events are also features of other group activities (22–24). Without an activity comparison, their respective contributions cannot be isolated. Singing, dance, and calligraphy are therefore useful settings for future comparison rather than activities to which the present account can already be generalized.

Pre-existing networks and selective participation provide further explanations. R06 entered through a former colleague; R05 combined prior skill with a family connection to the university and event contacts; R08 described longstanding sociability and work networks. These resources may have supported both participation and favorable accounts. Retained members are also more likely than absent leavers to describe arrangements that remained workable. Comparison with newcomers, people outside existing networks, and those who decline or discontinue participation is needed to assess these alternatives.

Migration-related comparison remains exploratory and concerns only R05 and R08. R05 described moving from Hunan after retirement to help care for a grandchild, having learned Tai Chi before relocation. R08 described long-term paid domestic care work in Beijing and participation arranged around employment. Only R05’s account clearly places relocation around retirement age; R08’s age at relocation was not directly elicited. Their differing circumstances direct attention to prior competence, existing ties, and care or work commitments rather than a uniform migrant experience. A general explanation of later-life migrant inclusion would require research designed around that population, with comparable migration histories and varied opportunities for participation.

### 4.6 Implications for community provision

The study’s relevance for public health lies in identifying the conditions under which community practice offers usable opportunities for contact and belonging. R05’s and R08’s scheduling conflicts, R04’s selective refusal of demanding material, contrasting views of paid lessons, and reported restrictions on shared space identify concrete questions for provision. Table 4 links these reported conditions to possible organizational responses and evaluation questions. The proposed responses are not interventions tested by this study.

**Table 4:** Reported participation conditions, proposed responses, and evaluation questions.

Reported condition	Interview basis	Proposed response requiring evaluation	Question for evaluation
Care and work schedules	R05 used an earlier practice site; R08 fitted practice around paid work.	Discuss alternative times and flexible involvement.	Can people with care or work commitments attend, including those currently absent?
Learning demands and performance pressure	R04 declined a difficult routine; R02 and R06 described anxiety or public evaluation.	Offer optional routines and event participation.	Can members sustain involvement without unwanted demands?
Teaching costs and continuity	R02 valued paid instruction; R04 questioned fees and continuity.	Clarify costs, teaching plans, and voluntary participation.	Who can access teaching, and how do views differ among participants and nonparticipants?
Permission and competing uses of space	R04 and R09 described restricted facilities; interviews also identified weather and noise constraints.	Co-design access and booking procedures with responsible partners.	Does usable access improve while accommodating other groups and residents?
Volunteer responsibilities and shared tasks	R01 undertook extensive coordination; R02 described substitute leaders and shared notices.	Discuss shared responsibilities and continuity arrangements.	Are responsibilities sustainable and open to members with different capacities?

Interview evidence supports the reported conditions. Organizational responses and evaluation questions are proposals, not demonstrated interventions or clinical guidance.

Inclusive provision should consider who attends and who remains unable or unwilling to participate. It should also consider whether volunteers can sustain their responsibilities. New contacts, team membership, institutional identification, residential attachment, and health outcomes should be assessed separately. Clinical assessment, movement adaptation, injury response, and referral arrangements require relevant professional expertise and a separate evidentiary basis. These interviews do not support prescribing screening tools, training doses, or insurance arrangements.

Co-design with practitioners, nonparticipants, community staff, and university partners could establish whether alternative times, optional activities, transparent teaching arrangements, or clearer access procedures address the reported constraints. Subsequent evaluation should examine changes in participation opportunities and participants’ experiences before attributing benefits to the activity or extending the model to other settings.

### 4.7 Strengths and limitations

The study’s strength lies in comparing different forms of involvement within a shared setting and retaining accounts that qualify the main explanation. The coding record permits selected passages to be traced through categories, while full-text checks distinguish participants’ statements from compressed summaries and interviewer formulations. The community worker’s perspective also contextualizes resource access without being treated as a substitute for residents’ experience.

Several limitations constrain interpretation. The case concerns one university-affiliated community with established networks and access to specialist expertise. Recruitment through the leader and routine practice favored established, cooperative participants; no comprehensive approach/refusal log or direct accounts from leavers, rejected applicants, or people unable to attend were available. Only two interviewees, R05 and R08, are identified as internal migrants. Their family-care and paid-work contexts differ, their migration chronologies are unevenly detailed, and only R05’s account clearly places relocation around retirement age. These accounts cannot establish population-wide migrant inclusion. Retrospective self-reports cannot demonstrate a common temporal sequence, causal effects, or community-wide change. Prior relationships and affirmative prompts may have shaped accounts, and no independently coded observation or formal member checking was available. Transcript normalization may have removed features of spoken expression; software-assisted English renderings were checked against edited Chinese textual context rather than independently validated against all recordings or through expert back-translation. Future work should follow varied participants over time, include people outside existing networks, and compare activities and settings with different resources.

## 5 Conclusion

This qualitative case shows how community Tai Chi can be experienced simultaneously as bodily learning, an everyday routine, and a setting for social participation. Its central contribution is to distinguish the connections among these experiences from an assumed progression toward community integration. Recurring practice offered opportunities for mutual notice and companionship, but practice acquaintances, team membership, university identification, and residential attachment remained different forms of involvement and belonging. The discrepant accounts are integral to this explanation: new acquaintances could coexist with a rejection of increased cohesion, and focal-team belonging could coexist with limited contact at another site.

The resulting embodied, relational, and place-based framework locates belonging in the interaction between practice and the conditions of participation. Instruction, volunteer coordination, and shared activities were valued alongside constraints involving care, paid work, costs, performance expectations, and institutional permission. The contrasting accounts of R05’s family caregiving after relocation and R08’s long-term paid domestic care work illustrate how shared practice may offer contact and team belonging across different living and working circumstances. Shared skill may offer a portable social credential for initial contact, but the evidence does not establish lasting inclusion, a common migration pathway, or an effect independent of group organization.

For active aging and community public health, the practical implication is to examine whether provision fits people’s responsibilities, preferences, and access to resources. Attendance and public visibility provide incomplete indicators of inclusion. Organizational proposals should be co-designed and evaluated in terms of who can participate, what relationships develop, which settings become meaningful, and whether volunteer responsibilities remain sustainable. Longitudinal and comparative research is needed to assess these connections and any health effects beyond this bounded community case.

## Data Availability

The interview transcripts and recordings are not publicly available because they contain personal and contextual information that could identify participants or third parties in this small community. De-identified supporting evidence is provided in Appendices A–C. Requests for additional redacted excerpts should be directed to the first author, Lei Zhang (202431070006@mail.bnu.edu.cn). Any access is subject to participant consent, applicable ethical requirements, and assessment of re-identification risk; identifiable transcripts and recordings will not be shared.
