# Supplementary material for: Tai Chi practice and community belonging in active aging: embodied, relational, and place-based embedding in a Beijing community
Source: Front Public Health. 2026 Oct 30;14:1986641. doi: 10.3389/fpubh.2026.1986641 (PMC13760686; doi:10.3389/fpubh.2026.1986641)
Supplement: Supplementary file 1 [file Supplementary_file_1.docx]

# Supplementary material

# Appendix A: Interview topics;

This topic summary is reconstructed from the recorded questions in the nine edited interview texts. It is provided for transparency and is not a verbatim pre-fieldwork interview guide. Questions and follow-ups varied by role. Some original questions contained affirmative assumptions; the analysis treats their wording as part of the interview context.

## Background and participation

Age and former or current work; first learning Tai Chi; time with the team; usual frequency and setting.

## Entry and alternatives

Why Tai Chi was chosen; other activities considered; how the person encountered and joined the activity.

## Bodily and emotional experience

Perceived changes in movement, daily routine, sleep or feelings; distinction between solo and group practice.

## Learning and difficulty

Difficult movements; instruction and video use; time spent practicing; discomfort or activities not undertaken.

## Relationships and belonging

New acquaintances; topics of conversation; whom the person could approach; whether participation affected community cohesion.

## Mobility and work where relevant

Residential move history; participation at other sites; family care and work schedules; limits on joining activities.

## Organization and events

Practice arrangements; leader and other members’ roles; competitions, demonstrations, qualifications and publicity.

## Resources and constraints

Space, instruction, funds and access; support received; perceived gaps; noise and competing uses.

## Future wishes and governance

Hopes for development and wider participation; the community worker’s account of projects, access, planning and anticipated scale.

Interpretive note: the reconstructed topics include questions about benefits and harmony because they were asked, not because those outcomes were assumed to occur. Participation in teaching or events and research recruitment are separate processes.

# Appendix B: Coding overview and analytic examples;

The coding record retains 95 selected units, 31 subcategories, and eight main categories. Table B1 shows how checks against full interview context changed selected interpretations. Earlier initial labels are translated from the coding record and are included to make those changes visible; they are not all endorsed as current findings. Table B2 lists the subcategories with representative paraphrases and qualifications. Unit identifiers retain correspondence with the internal record; identifying filenames are omitted.

Table B1. Examples linking evidence, initial labels, categories, and revised interpretation

| **Evidence checked in context** | **Earlier initial label** | **Code path** | **Revised interpretation** |
| --- | --- | --- | --- |
| R02 distinguished practice friends from close friends while describing mutual notice (U019). | Weak ties among practice friends provide everyday care. | E1 → E | Retain recurring contact and support; do not infer intimacy or residential belonging. |
| R05 learned in 2019 and moved in 2023; the earlier move was his spouse’s (U047). | Care-related relocation and the possibility of solo practice initiate participation. | A2 → A | Correct the chronology: learning preceded his own relocation. Treat prior skill as an entry resource. |
| R08 watched and copied movements near work but said she had not really joined that group (U082). | Shared skill lowers the threshold for integration across communities. | E2 → E | Narrow integration to a contact opportunity; distinguish it from belonging to the focal team. |
| R08 recounted a care recipient’s injury and his own attribution to intensive practice (U084). | Excessive training produces adverse health outcomes. | H3 → H | Report a second-hand safety concern; withdraw the clinical causal interpretation. |

*Note: Code paths identify the subcategory and main category in the retained record. Evidence is paraphrased here; selected original Chinese passages and English translations are provided in Appendix C.*

Table B2. Overview of the 31 subcategories

| **Subcategory** | **Representative paraphrase and unit IDs** | **Interpretive qualification** |
| --- | --- | --- |
| A1 Health concerns and recovery | R02 sought a suitable activity because of discomfort; R04 searched after a health episode. U013, U038 | Perceived motivation, not treatment benefit |
| A2 Retirement and care transitions | R05 learned in 2019 and moved in 2023; R08 gained time after a change in care work. U047, U076, U083 | R05's relocation chronology is documented; R08's age at relocation was not directly elicited |
| A3 Activity fit and preference | R03 valued being able to practice alone; R06 initially resisted the group. U028, U058 | Preference and peer encouragement can coexist |
| B1 Perceived function and health | R06 associated routine with feeling that life had greater quality. U064 | No clinical assessment |
| B2 Attention and emotional experience | R02 described concentration; R08 described absorption and relaxation. U014, U075 | Subjective experience, not a measured psychological mechanism |
| B3 Routine and perceived competence | R04 treated practice like work; R05 described an established habit. U039, U048 | Does not establish later social or place outcomes |
| B4 Appearance and self-presentation | R05 valued Tai Chi clothing; R06 avoided attracting attention. U054, U065 | Aesthetics can attract and deter |
| C1 Learning difficulties | R06 struggled to coordinate hands and feet. U059 | No objective learning comparison by age |
| C2 Demonstration and correction | R02 valued instruction; R08 described clarification of a disputed fan movement. U016, U079 | Perceived value; not verified injury prevention |
| C3 Video review and practice | R03 combined instruction, video review, and personal experimentation. U029 | Interview description; no analyzed video-use records |
| C4 Event-related learning | R02 rehearsed intensively and felt anxious; R07 valued awards. U015, U073 | Performance did not suit all participants |
| D1 Volunteer organization | R01 learned in order to teach; R04 valued her coordination. U004, U041 | Responsibilities concentrated; some substitutes also reported |
| D2 Recurring schedules and mutual notice | R02 connected a group routine with persistence; R07 differentiated solo and collective practice. U017, U068 | No uniform attendance or tested adherence effect |
| D3 Welcome, mutual help, and norms | R01 welcomed different backgrounds but preferred shared values; R06 recalled disagreement. U008, U012, U062 | Potential informal boundary; no rejected applicant interviewed |
| D4 Linked learning and events | R01 discussed varied activities; R04 valued having choices. U009, U040 | Cycle partly formulated in interview dialogue; no verified feedback loop |
| E1 Practice acquaintances | R02 called peers practice friends; R07 knew more people but denied increased cohesion. U019, U070 | Acquaintance and community cohesion are distinct |
| E2 Contact across sites and migration histories | R05 joined a park group after relocation for grandchild care; R08, a migrant domestic care worker, expressed focal-team belonging but limited contact at another site. U050, U078, U082 | Site mobility is not residential migration or completed integration |
| E3 Family and work-network diffusion | R08 practiced with care-worker peers and shared videos with family. U077, U081 | Partly extends existing ties; others’ participation not independently verified |
| F1 Nearby specialist knowledge | R03 valued expert explanation; R09 hoped for further university collaboration. U037, U091 | Existing teaching and proposed partnerships are distinct |
| F2 Association contacts and opportunities | R01 sought training, events, and exchange through association links. U011 | Plans and opportunities do not establish participation outcomes |
| F3 Community project support | R01 reported funding and materials; R09 explained applications and accountability. U006, U010, U087, U088 | Reported support; no causal attribution to reputation |
| F4 Permission and resource coordination | R04 and R09 described difficulty using university-owned space. U042, U089 | Perceived barriers, not a systematic access audit |
| G1 Interpreting movement culturally | R01 related opening and closing to managing everyday pressure. U005 | Participant interpretation rather than a verified therapeutic mechanism |
| G2 Clothing, music, and visible style | R01 valued coordinated appearance; R02 distinguished Tai Chi from other activities. U003, U023 | Status judgments require reflexive interpretation |
| G3 Public and digital visibility | R05 described sharing event videos and passers-by asking about practice. U055, U056 | Interest and visibility are not measured recruitment |
| G4 Collective pride and branding | R01 recalled university representation; R09 proposed community branding. U002, U092 | Experienced team pride versus an intended governance outcome |
| H1 Space, weather, and noise | R02 described a crowded setting; R07 wanted suitable winter space. U021, U072 | Reports of constraints, without direct interviews with complainants |
| H2 Time and demographic mismatch | R05 used an earlier site; R08 worked during daytime practice. U049, U063, U090 | Retained participants describe barriers; nonparticipants absent |
| H3 Safety concerns and limiting demands | R04 declined new material; R07 feared injury; R08 described a client’s knee problem. U046, U069, U084 | Second-hand injury attribution is not a clinical finding |
| H4 Reliance on people and resources | R04 described substantial organizer work; R02 questioned tangible support. U026, U045 | Uneven perceived support; no test of succession or funding models |
| H5 Possible management problems with growth | R09 contrasted managing 20 and 200 people as a hypothetical example. U094 | Anticipated governance burden, not observed expansion |

*Note: Category prefixes A–H correspond to Table 2. Display labels are descriptive renderings that reflect the revised interpretation. Counts retained for A–H are 12, 8, 9, 12, 14, 11, 11, and 18, respectively (total 95). They describe the selected record, not the frequency or strength of findings in the full corpus.*

# Appendix C: Evidence checks and interpretive revisions.

The excerpts below preserve qualifications relevant to the explanation. Square brackets clarify context; ellipses indicate omissions. Paraphrases and third-party reports are identified. Unit IDs refer to the retained coding record; contextual checks draw on the full edited interviews. Participant characteristics and migration contexts are summarized in Table 1.

## C1 R05 Learning before family-care relocation

“I retired in 2023 and came to live here [near the university].” “I enrolled in a Tai Chi class in the second half of 2019.”
“I asked, ‘May I join you?’ They said yes.” “I feel that knowing a little Tai Chi helps me find friends quickly.”

The learning and relocation account is linked to U047–U050. R05 was a retired teacher from Hunan who moved to Beijing after retirement to help care for a grandchild while his adult child worked there. His 1963 birth year and 2023 move imply relocation at approximately age 60. The earlier U047 summary incorrectly conflated his spouse's earlier move with his own; his systematic learning preceded his relocation. His main practice site was elsewhere, and event invitations and a university family connection also facilitated access. In additional interview context, R05 described friends who had moved to other cities for grandchild care and found Tai Chi groups there. Those are second-hand examples reported by R05, not additional interviewees, independently verified integration outcomes, or additional units in the 95-unit record.

## C2 R08 Paid domestic care work and belonging across sites

“It feels like I have found a group to belong to.” “I have not really joined their group; I just watch and copy some movements.” “It is working time, after all; work comes first.”

The first statement concerns the focal team; the statements about watching, copying, and prioritizing work concern another practice site near work (U078, U082–U083). R08 was an internal migrant undertaking long-term paid domestic care work in the university-affiliated community and reported 16 years in Beijing. Her occupational description is that of a paid domestic care worker, not a retired teacher or a person relocated solely for family reunion. Her exact relocation date and age at relocation were not directly elicited. The additional excerpt shows how current participation depended on available personal time. Reports about other domestic care workers provide occupational context rather than an independently sampled group. The contrast supports focal-team belonging alongside limited second-site contact, not a generalized migrant-integration outcome. Prior sociability and work networks remain alternative explanations.

## C3 R07 Acquaintance without a cohesion claim

Question: Has practicing Tai Chi helped increase community cohesion?
Answer: “No; I just come to practice.”
“Before practicing Tai Chi, I did not know most of these people.”

U070 and its full conversational context. R07 also described occasional disclosure to people she trusted. The negative case therefore qualifies the link from acquaintance to community cohesion; it does not negate all relationship support. This comparison supports separable dimensions instead of required stages.

## C4 R04 Declining part of the program

“They started learning another new routine, but I did not join in. I could not manage more than 50 new movements.”

U046 and surrounding account. This is a direct example of declining a component while remaining a practitioner. It supports variation in preferred demands, not an age-specific prescription or an account of leaving the team.

## C5 R08 Injury account about another person

“He had a problem with the meniscus in his knee…” “He practiced too intensely, he himself said…”

U084. R08 reported a care recipient’s account, including his own attribution. No record of clinical assessment, exposure, or causal investigation was provided. We therefore report a second-hand safety concern; the account does not establish that intensive Tai Chi caused the injury. R06’s own discomfort is a separate first-hand report, also without clinical verification.

## C6 R09 Plans and a hypothetical scale comparison

“Our current plan is first to make it a recognizable community program…”
“For example, managing 20 people before would certainly differ from managing, say, 200 in the future.”

U092 and U094. The first is a plan, and the second a hypothetical warning. Neither establishes an achieved community brand, observed growth to 200, or breakdown after expansion.

## C7 R02 The meaning of practice friends

“I would not call them friends; I would call them practice friends.”

U019. The surrounding passage describes questions about absence and health. We retain the limited relationship label while recognizing mutual notice. The term does not imply deep friendship or residential integration.

## Additional comparison decisions

R01’s stated welcome (U008) was read alongside preferences for compatible values (U012), revealing a possible informal entry boundary without documenting an actual refusal. R02’s appreciation of optional paid lessons was compared with R04’s concern about their continuation. R02 also described substitute practice leaders, qualifying an absolute single-leader-dependence claim. The learning–practice–event cycle (U009) was reclassified as a summary partly developed through interviewer prompting. These contextual checks refine the interpretation without claiming fresh interviews, recoding reliability, or a new saturation test.
